# In Vitro Assays for the Assessment of Impaired Mitochondrial Bioenergetics in Equine Atypical Myopathy

**DOI:** 10.3390/life11070719

**Published:** 2021-07-20

**Authors:** Caroline-J. Kruse, David Stern, Ange Mouithys-Mickalad, Ariane Niesten, Tatiana Art, Hélène Lemieux, Dominique-M. Votion

**Affiliations:** 1 Department of Functional Sciences, Faculty of Veterinary Medicine, Physiology and Sport Medicine, Fundamental and Applied Research for Animals & Health (FARAH), University of Liège, 4000 Liège, Belgium; Tatiana.Art@Uliege.be; 2 Equine Pole, Fundamental and Applied Research for Animals & Health (FARAH), Faculty of Veterinary Medicine, University of Liège, 4000 Liège, Belgium; d.stern@Uliege.be (D.S.); dominique.votion@Uliege.be (D.-M.V.); 3 Center for Oxygen, Research & Development (CORD), Center for Interdisciplinary Research on Medicines (CIRM), Institute of Chemistry, B6a, University of Liège, Allée du Six Août, 11, 4000 Liège, Belgium; amouithys@Uliege.be; 4 Center for Oxygen, Research & Development (CORD), Fundamental and Applied Research for Animals & Health (FARAH), Institute of Chemistry, B6a, University of Liège, Allée du Six Août, 11, 4000 Liège, Belgium; Ariane.Niesten@Uliege.be; 5 Faculty Saint-Jean and Department of Medicine, University of Alberta, 8406-91 Street, Edmonton, AB T6C 4G9, Canada; helene.lemieux@ualberta.ca

**Keywords:** atypical myopathy, high-resolution respirometry, toxicity assays, cell culture, equine primary myoblasts

## Abstract

Equine atypical myopathy is a seasonal intoxication of grazing equids. In Europe, this poisoning is associated with the ingestion of toxins contained in the seeds and seedlings of the sycamore maple (*Acer pseudoplatanus*). The toxins involved in atypical myopathy are known to inhibit ß-oxidation of fatty acids and induce a general decrease in mitochondrial respiration, as determined by high-resolution respirometry applied to muscle samples taken from cases of atypical myopathy. The severe impairment of mitochondrial bioenergetics induced by the toxins may explain the high rate of mortality observed: about 74% of horses with atypical myopathy die, most within the first two days of signs of poisoning. The mechanism of toxicity is not completely elucidated yet. To improve our understanding of the pathological process and to assess therapeutic candidates, we designed in vitro assays using equine skeletal myoblasts cultured from muscle biopsies and subjected to toxins involved in atypical myopathy. We established that equine primary myoblasts do respond to one of the toxins incriminated in the disease.

## 1. Introduction

Equine atypical myopathy (AM) is a severe environmental intoxication linked to the ingestion of certain maple (*Acer*) seeds and seedlings. In Europe, the incriminated tree is the sycamore maple (*Acer pseudoplatanus*) [1], whereas in the United States, the box elder (*Acer negundo*) has been linked to the poisoning [2]. Two toxins, hypoglycin A (HGA) [3,4,5] and methylenecyclopropylglycine (MCPrG) [6] are involved in the poisoning [7]. These molecules are not toxic per se but once in the body are transformed into their active metabolites, methylenecyclopropylacetyl-CoA (MCPA-CoA) and methylenecyclopropylformyl-CoA (MCPF-CoA), respectively [8,9,10]. Both toxins are known inhibitors of fatty-acid ß-oxidation, which results in an impaired capacity of energy production using oxidative metabolism [10,11,12,13]. The MCPA-CoA inhibits also dehydrogenases involved in the degradation of branched-chain amino acids [10]. The ingestion of maple toxins led to the detection of toxins, conjugated toxic metabolites, and fatty esters in blood [1,2,5,7,14,15,16,17]. 

Clinical signs in intoxicated horses include muscle weakness and stiffness, eventual recumbency, and, in 74% of cases, death [18]. Macroscopic, histologic, and histochemical analyses confirm multifocal degeneration and necrosis with variable severity between cases and muscles [19]. Indeed, muscular lesions seem to be more constant and severe in the myocardium and respiratory and postural muscles [19,20], therefore, oxidative muscle fibers appear to be particularly affected by the toxin [19]. Transmission electron microscopy revealed several ultrastructural changes affecting especially mitochondria, as matrix loss and cristae fragmentation [19].

Previous studies performed on skeletal muscle show that structural alterations are associated with mitochondrial functional consequences [13]. Using high-resolution respirometry (HRR), a severe depression of mitochondrial oxidative phosphorylation (OXPHOS) and electron transfer system capacities (ET capacity) in AM affected horses was found [13] using standardized substrate-uncoupler-inhibitor titration (SUIT) protocols validated for respirometric assessments of equine muscle cells [21].

Since AM outbreaks are seasonal (i.e., autumnal and spring outbreaks resulting from the consumption of seeds and seedlings, respectively) and do not occur to the same extend every year [22], in vivo sampling is naturally limited. Also, because of the acute nature of AM and the rapid progression of the condition with a mean survival time of 38 h [23], complementary examinations and sampling might be difficult to perform. Additionally, the use of surrogate animals to study AM may not be valid, since both rodents and rabbits display damage of different organs than horses after HGA intoxication and do not show signs of rhabdomyolysis [24,25]. Because of these obstacles, an in vitro model was designed attempting to reproduce mitochondrial dysfunction by adding methylenecyclopropylacetyl (MCPA) (i.e., the sole toxin commercially available) during HRR experiments. Ultimately, our final goal will be to define a treatment for AM based on the ability of the drug to restore an adequate mitochondrial function. The aim of the present study was: (1) to reproduce specific changes in OXPHOS capacity and respiratory control patterns observed in skeletal muscle of AM affected horses using conventional SUIT protocols [13]; (2) to measure the effect of MCPA on fatty acids utilization, and (3) to determine the cytotoxicity and viability of equine primary muscle cells subjected to HGA and MCPA.

## 2. Materials and Methods

### 2.1. Cell Culture of Equine Primary Myoblasts

Equine primary skeletal myoblast cultures were purchased from RevaTis^®^ (RevaTis, Aye, Belgium). A vial containing 2.5 million cells was thawed at 37 °C in a water bath. The cells were then cultivated in an 80 cm^2^ culture Tflask and multiplied from passage 5 to passage 8. All manipulations were performed under streamline flow hood. 

Cells were cultured in the maintenance media Dulbecco’s modified Eagles’s low-glucose DMEM 1 g/L (Lonza, Verviers, Belgium) supplemented with 20% fetal bovine serum, 1% L-glutamine, 1% penicillin-streptomycin, and 0.5% amphotericin B (Thermo Fisher Scientific, Karlsruhe, Germany). When 80% confluence was reached, culture medium was removed, and the flasks were washed using Dulbecco’s phosphate-buffered saline (DPBS) without Ca^2+^ and Mg^2+^. Cells were subsequently trypsinized (TrypLE^TM^ Express, Thermo Fisher Scientific, Karlsruhe, Germany), centrifuged, and counted according to Ceusters et al. [26]. The equivalent of one 175 cm^2^ culture T-flask was used to continue cell culture to further passages.

At each passage, part of the cells was immediately processed for HRR. Ten thousand cells per well were seeded in a 96 clear bottom white plate (Cellstar^®^ Greiner Bio-One, Vilvoorde, Belgium) and supplemented with DMEM medium until further analysis. Plates and flasks were incubated at 37 °C and 5% CO_2_ in a humidified incubator.

### 2.2. Toxicity Assays 

The cell toxicity of MCPA was assessed using the CellTox^TM^ Green assay (Promega Benelux, Leiden, The Netherlands). This assay was developed to determine toxic effects by binding DNA of cells with impaired membrane integrity. 

Plated cells were kept in a humidified incubator at 37 °C and 5% CO_2_ until 80% of confluence was reached. Confluence was assessed by light microscopy after 24 to 36 h. Once confluent, the cells were washed with DPBS without Ca^2+^ and Mg^2+^. The cells were then exposed to different concentrations of MCPA (Merck, Darmstadt, Germany) with DMSO as solvent and HGA (Toronto Research Chemicals, Ontario M3J2K8, Canada) suspended in DMEM medium without phenol red. CellTox^TM^ Green dye was added to each well. The bottom of the plate was covered with a BrightMax^TM^ seal (Greiner Bio-One, Vilvoorde, Belgium) and subsequently placed in the EnSpire^®^ Multimode Plate Reader (PerkinElmer, Waltham, MA, USA) for reading. Data was recorded for 24 h after toxin exposure in order to determine the evolution of cytotoxicity.

Additionally, a real-time viability assay through reducing potential measurement was performed. The nonlytic dye contained in the RealTime-Glo^TM^ MT Cell Viability Assay (Promega Benelux, Leiden, The Netherlands) allows for continuous reading and is based on the continuous reduction of the viability substrate by the viable cells contained in each well. The DMSO concentrations in each well were of 0.5% in order to exclude DMSO induced toxicity to the cells. 

### 2.3. High-Resolution Respirometry

After centrifugation, the cells were counted and then placed in MiR05 mitochondrial respiration medium (0.5 mM EGTA, 3 mM MgCl_2_. 6 H_2_O, 20 mM taurine, 10 mM KH_2_PO_4_, 20 mM HEPES, 1 g/L BSA, 60 mM potassium-lactobionate, 110 mM sucrose, pH 7.1) at 37 °C. In total, 2.5 to 3 million cells were added to each 2 mL Oxygraph-2k chamber (Oroboros Instruments, Innsbruck, Austria). Oxygen concentration (μM) and oxygen flux per million cells (pmol O_2_∙s^−1^∙10^6^ cells^−1^) were recorded online using DatLab software (Oroboros Instruments, Austria). Experiments were conducted using specific SUIT protocols and oxygen levels were maintained between 200 and 500 μM O_2_ to avoid any oxygen-related limitations of respiration and to align with previous studies [13,21,27]. Oxygen flux was expressed as respiration per million cells (pmol O_2_∙s^−1^·10^6^ cells^−1^), or as control ratios, namely flux control ratios (*FCR*). These ratios have the advantage of being independent of cell count, mitochondrial content, and density, indicating qualitative changes of mitochondrial respiratory control [28].

**Figure 1:**
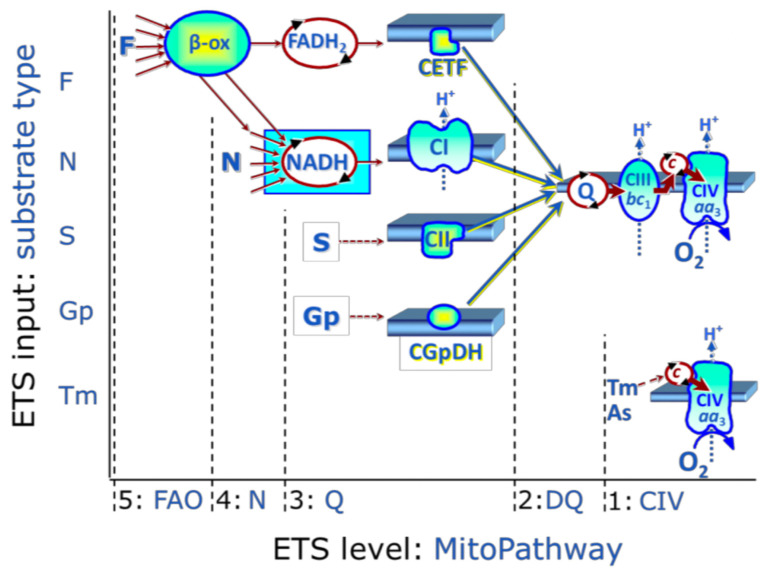
ET-substrate types are linked to ET-pathway types in substrate-uncoupler-inhibitor titration (SUIT) protocols. Electrons from multiple upstream origins feed into the Q junction. These origins include (5) fatty acid ß-oxidation (FAO) providing electrons from FADH_2_ to the electron-transferring flavoprotein complex (CETF; F pathway), (4) dehydrogenases and enzymes converging at the N-junction and providing electrons from NADH to complex I (N pathway), (3) succinate (S) providing electrons from FADH_2_ to Complex II (CII; S pathway). From the Q junction, electrons are then transferred to Complex III, cytochrome c and complex IV, before their transfer to O_2_ to form H_2_O. Figure from Gnaiger (2020) with permission [28]. Abbreviations: FADH_2_ = flavin adenine dinucleotide; NADH = Nicotinamide adenine dinucleotide.

Several SUIT protocols were applied, using both fatty acids and reduced nicotinamide adenine dinucleotide (NADH)-linked substrates in order to assess mitochondrial pathways at different levels of integration. In order to have a global overview, the electron transfer (ET) pathway was fed by the NADH junction (N-junction) substrates pyruvate, glutamate in the presence of malate. Fatty acids are catabolized via ß-oxidation and support ET pathways through both reduced flavin adenine dinucleotide (FADH_2_) junction (F-junction) and N-junction (see level 5; Figure 1). Therefore, ß-oxidation relies on the combination of F-junction and N-junction pathways. 

Muscle cells suspended in MiR05 were added to the Oxygraph-2k chambers containing a final volume of 2 mL per chamber maintained at 37.0 °C. In SUIT1, electron flow was sustained by the NADH-linked substrate glutamate and co-substrate malate (GM; 10 and 2 mM, respectively) followed by a saturating concentration of ADP (D; 2.5 mM). In SUIT2, the initial substrate was pyruvate with malate as co-substrate (PM; 5 and 2 mM, respectively) with subsequent addition of digitonin (Dig; 10 µg∙10^6^ cells^−1^) and ADP (D; 2.5 mM) followed by addition of glutamate (G; 10 mM). Three fatty acids’ protocols were performed and started with the addition of acetylcarnitine (Act; 5 mM; SUIT3), octanoylcarnitine (Oct; 0.5 mM; SUIT4), and palmitoylcarnitine (Pal; 0.04 mM; SUIT5), and malate (2 mM; SUIT3, SUIT4, SUIT5) as co-substrate. In SUIT3 to 5, electrons from both the electron-transferring flavoprotein complex (CETF) and the Complex I (CI) entered the Q-junction. In all protocols, digitonin (Dig; 0.01 mM) was added before ADP. Optimal digitonin concentration was determined by careful titration experiments as previously described [29]. In all SUIT protocols, ADP-stimulated respiration represents OXPHOS capacity, *P* whereas ET capacity, *E* was obtained by addition of the uncoupler FCCP, rendering this state as not limited by the capacity of the phosphorylation system [28].

In every protocol, succinate (S; 10 mM) was subsequently added for electron flow from Complex II (CII) into the Q-junction to give the flux through the NS-pathway (N and S pathway combined) or the F- and S-pathway combined. By stepwise addition of the non-physiological uncoupler FCCP (U; 0.05 μM, followed by 0.025 μM steps until maximal oxygen flux was reached), ET-capacity, *E*, was obtained. Electron input into the Q-junction through CII only was subsequently measured by inhibition of CI with rotenone (Rot; 0.5 μM). Finally, residual oxygen consumption state was obtained by addition of antimycin A (Ama; 2.5 μM) to inhibit Complex III (CIII). For each protocol, MCPA [9 mM] was added to a parallel chamber after ADP addition. The concentration was defined beforehand by toxicity assays. All protocols are summarized in Table 1.

For all protocols, a second oxygraphic run was performed to establish if cytochrome *c* (Cyt *c*; 10 µM) addition induced an increase in O_2_ flux. Cytochrome *c* release is considered as an essential quality control because of the possible limitation of active respiration when the outer mitochondrial membrane has been damaged by the laboratory procedures, allowing the loss of cytochrome *c* located in the intermembrane space [28].

### 2.4. Total Protein Content

Total protein content was measured using the Pierce^TM^ 660 nm Protein Assay. Absorbance was recorded using a spectrophotometer and MPM6 analysis software. Measurements were performed for each passage of one vial in duplicate (N = 1, n = 2).

### 2.5. Data Analysis

Raw respirometric data was normalized by *FCR* to allow direct comparison with the results presented in Lemieux et al. [13]. The pathway control ratios of oxygen flux with substrate types provided separately for the N- and S-pathways were normalized for flux through the combined NS-pathway (i.e., N/NS and S/NS). All respiratory states were corrected for residual oxygen consumption. For treated cells, MCPA was added after ADP and raw data were collected after MCPA addition. The different nominators and denominators were calculated by subtracting residual oxygen consumption from every calculated raw data. Regarding our ratios, CI*_P_* and CI + CII*_P_* are relative to OXPHOS capacity whereas CI + CII*_E_* and CII*_E_* are relative to ET capacity. Also, it is assumed that S-pathway linked respiration is not influenced by uncoupling [13,21] and therefore CII*_E_* = CII*_P_*. The ratios obtained for SUIT1 and SUIT2 in MCPA-treated cells were compared to ratios calculated in MCPA affected horses. Two vials from the same horse were used for respirometric analysis and runs were performed in duplicate (N = 2, n = 2), when not specified otherwise. Additionally, for each raw parameter in MCPA treated cells, a percentage from the control cells was calculated. Similarly, the effect of MCPA was evaluated with SUIT3, SUIT4, and SUIT5.

For a same respirometric parameter, a two-tailed *t*-test was performed between control and treated cells. Statistical significance was set at *p* < 0.05. In the tables, means are represented ± standard error of the mean (SEM). Additionally, ratios of MCPA treated cells were compared to previously published ratios of AM affected horses in Lemieux et al. via unpaired two-tailed *t*-test. For this comparison, ratios of different passages were pooled. 

Regarding toxicity assays, data were analyzed by GraphPad Prism and a non-linear regression (sigmoidal, 4PL, least squares fit) was performed. For cell reduction potential, a non-linear regression (sigmoidal, 4PL, least squares fit) was also performed for each tested MCPA concentration at different times.

## 3. Results

### 3.1. Toxicity Assays 

**Figure 2:**
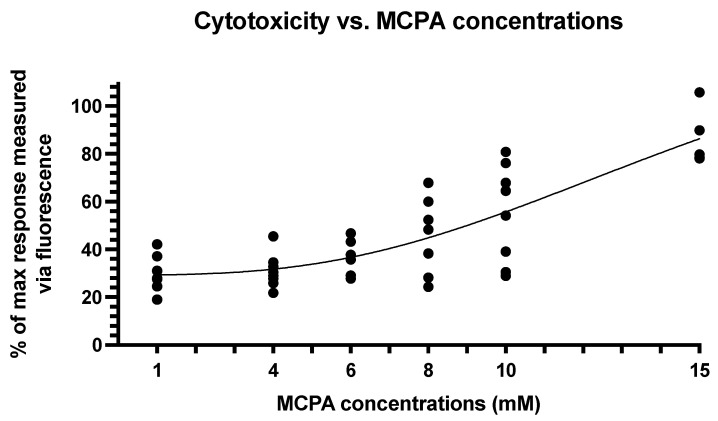
Methylenecyclopropylacetyl (MCPA) induced cytotoxicity measured by CellTox^TM^ Green.

When analyzed independently, the kinetics of recorded fluorescence show a steep augmentation reaching a plateau at approximatively 10 h after toxin exposure. Plateau values were grouped for each concentration. After a 24-h exposure, wells submitted to 15 mM MCPA displayed between 78 and 105% of maximal response compared to the lysis solution (Promega Benelux, Leiden, The Netherlands). An important increase of cytotoxicity was observed between 10 and 15 mM (Figure 2). Best-fit values for total data, regardless of the passage indicated an IC50 of 15.7 mM. This value should however be interpreted with caution since a complete confidence interval could not be calculated. Also, it is notable that the differences between individual passages do result in very different responses to MCPA. Indeed, cytotoxicity ranged widely from 25 to 67% for 8 mM and from 29 to 76% for 10 mM. Lowest toxicity was measured at passage 8 with 25 and 28% for 8 mM and 29 and 31% for 10 mM.

**Figure 3:**
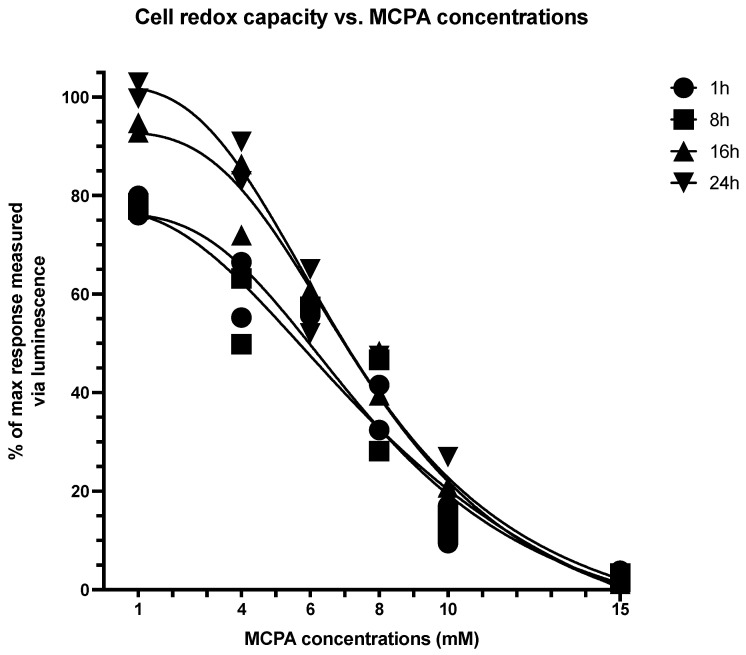
Cell reduction capacity depending on methylenecyclopropylacetyl (MCPA) concentrations at different time points.

Regarding cell reducing potential, a non-linear regression (sigmoidal, 4PL, least squares fit) was also performed for each tested MCPA concentration at different times. Best-fit values indicated an IC50 of 8.09 ± 0.55 mM depending on the time recorded (Figure 3). In neither toxicity assay, HGA addition to cell cultures at concentrations ranging from 0.25 to 1 mM resulted in changes compared to control cells (data not shown). According to best-fit values, MCPA addition for subsequent experiments (i.e., HRR) was set at 9 mM.

### 3.2. High-Resolution Respirometry

**Figure 4:**
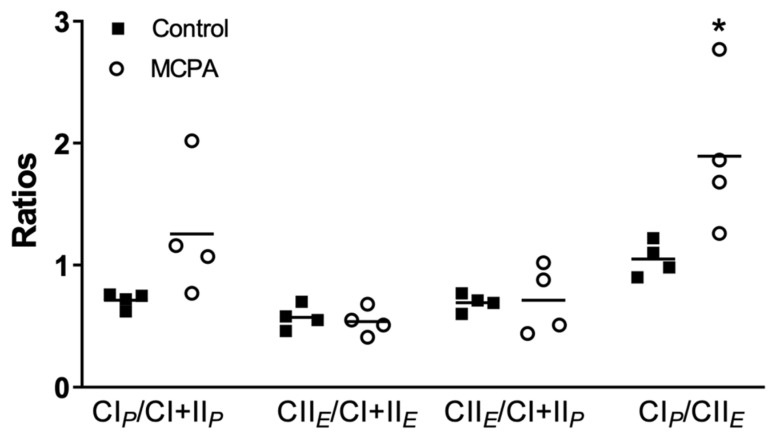
Ratios in control cells and cells treated with 9 mM methylenecyclopropylacetyl (MCPA) for the substrate-uncoupler-inhibitor titration protocol 1 (SUIT1). Each dot represents one passage, and the same passage are represented with or without MCPA. The bar represents the mean. * Significantly different from controls (*p* < 0.05) with a t-test were indicated with a *. Abbreviations: CI*_P_* = Complex I linked OXPHOS capacity; CII*_E_* = Complex II linked ET capacity; CI + CII*_P_* = Complex I&II linked OXPHOS capacity; CI + CII*_E_* = Complex I&II linked ET capacity.

At any passage, the addition of cytochrome *c* resulted in a slight (i.e., ≤10%) increase in O_2_ flux thus confirming the preservation of the outer mitochondrial membrane integrity. When comparing obtained *FCR* of control vs. MCPA treated cells in the SUIT1 protocol (with glutamate and malate), a significant difference was observed for the ratio CI*_P_*/CII*_E_*, which was increased with 9 mM MCPA (Figure 4). For the three ratios with CI + CII at the denominator, there were no significant changes with MCPA treatment.

**Figure 5:**
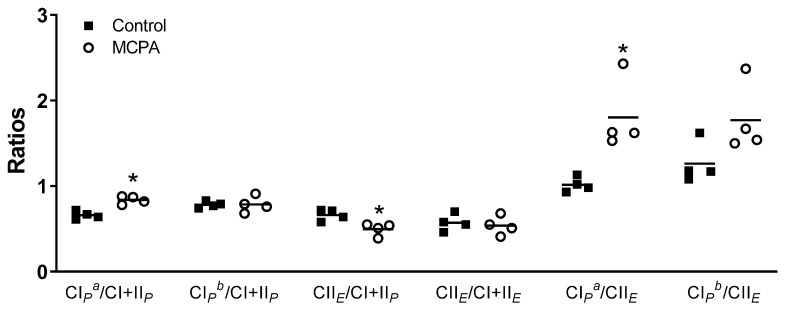
Ratios in control cells and cells treated with 9 mM methylenecyclopropylacetyl (MCPA) for the substrate-uncoupler-inhibitor titration protocol 2 (SUIT2). Each dot represents one passage, and the same passage are represented with or without MCPA. The bar represents the mean. * Significantly different from controls (*p* < 0.05) with a t-test were indicated with a *. ^a^ using glutamate and malate as N pathway substrates. ^b^ using pyruvate, malate and glutamate as N pathway substrates.

In SUIT2, significant differences between groups were also detected. Compared to the control cells, the MCPA treated cells showed an increase in the ratios of CI*_P_^a^*/CI + II*_P_* and CI*_P_^a^*/CII*_E_* and a decrease in the ratio CII*_E_*/CI + II*_P_* (Figure 5). Interestingly, the ratio CI*_P_*/CI + II*_P_* was significantly different between groups when pyruvate and malate were used as substrates, but not when glutamate was added (Figure 5). The ratio of CII/CI + II was significant only when the denominator (CI + II) was taken under the *P* state, but not when it was taken under the *E* state (Figure 5).

Without considering cell passages, a decrease in oxygen flux of 46% in average (SEM ± 0.08) was recorded after MCPA addition to the chamber in SUIT1. This decrease remained relatively constant for the rest of the protocol: CI + II*_P_* = 43% of control (SEM ± 0.15), CI + II*_E_* = 40% of control (SEM ± 0.2), CII*_E_* = 32% (SEM ± 0.13) (Table 2). Similarly, a decrease in oxygen flux to 53% of control cells (SEM ± 0.11) was recorded after MCPA addition to the chamber (CI*_P_*). A constant decrease was noted for the rest of the protocol: CI + II*_P_* = 43% of control (SEM ± 0.10), CI + II*_E_* = 40% of control (SEM ± 0.17), CII*_E_* = 29% (SEM ± 0.09). 

Similarly, for fatty acid protocols using acetylcarnitine as substrate, oxygen flux immediately after MCPA addition was on average 67% of the control value (SEM ± 0.2). When Succinate was added, 61% of control (SEM ± 0.24) and after FCCP addition 72% of control (SEM ± 0.08). Finally, after rotenone addition, 51% (SEM ± 0.12) of control were attained. When the fatty acid substrate was octanoylcarnitine, oxygen flux immediately after MCPA addition was on average 74% of the control value (SEM ± 0.24). For the rest of the protocol: CI + II*_P_* = 62% of control (SEM ± 0.14), CI + II*_E_* = 56% of control (SEM ± 0.24), CII*_E_* = 49% (SEM ± 0.15). Fatty acid protocols using palmitoylcarnitine as substrate, oxygen flux immediately after MCPA addition was on average 67% of the control value (SEM ± 0.07). For the rest of the protocol: CI + II*_P_* = 60% of control (SEM ± 0.11), CI + II*_E_* = 58% of control (SEM ± 0.31), CII*_E_* = 37% (SEM ± 0.25). Hence, a decrease in respiration was recorded in all SUIT protocols used. Also, whatever the protocol, initial LEAK respirometric parameters were similar between control and treated cells.

Regardless of the substrate sustaining electron flow used, the addition of MCPA depressed respiration in treated cells. This effect was noted at each cell passage. 

### 3.3. Total Protein Content

In order to compare macroscopic cell count to an internal measure, total protein content was analyzed (Table 3). Despite a similar cell count, the total protein content seemed to vary between runs (*no statistical analysis performed*). Since total protein content was not measured for each vial used, internal normalization by ratios was preferred. These ratios are independent of cell count or tissue mass [28].

## 4. Discussion

Regardless of the protocol, an immediate effect of MCPA on mitochondrial electron transfer system (ETS) complexes was observed. This is interesting as it corroborates the in vivo observations obtained in horses with AM [11]. Similar to findings in Lemieux et al. [13], mitochondrial respiration seemed to be more depressed with glutamate-malate sustained respiration (SUIT1) compared to pyruvate-glutamate-malate (SUIT2). Indeed, a severe depression of both OXPHOS and ET capacity could be reproduced in vitro. In all five SUIT protocols, the addition of MCPA resulted in an immediate effect on the N- and S-pathway but also on the F-pathway, sustained by fatty acid ß-oxidation. When analyzing SUIT2, the contribution of the S-pathway was similar to the N-pathway in affected and control horses [13]. However, in our study, the S-pathway seemed to be more affected and therefore resulted in a strong diminution of OXPHOS and ET capacity, no matter which protocol was used. This finding is probably linked to a longer exposure time to the toxin since CII-linked activity is measured after CI and CETF sustained O_2_ flux.

Regarding fatty acid substrates, it is also important to note that ß-oxidation supplies electron transfer through the N-junction as well as the rate-limiting F-junction pathway branches. Even though a progressive decrease in respiration was also recorded with SUIT3, 4 and 5, F-pathway combined with S-pathway sustained respiration resulted in better supported respiration than the NS-pathway. 

Overall, MCPA addition to the oxygraphy chamber resulted in a generalized inhibitory effect, acting either on all ETS complexes, or having a specific target in downstream ETS components as Q, CIII or CIV. In order to address this question, enzymatic assays testing either the individual complexes’ response to the toxin or related enzymes upstream (e.g., PDH) and other SUIT protocols targeting specific downstream complexes will need to be applied in future studies. Also, it cannot be excluded that the IC50 calculated on the basis of toxicity and viability assays was too high to identify the first target and resulted in a general decrease of O_2_ flux.

It is also noteworthy that despite the recording of a depression in mitochondrial respiration, without considering the passage and the cell batch, the differences between the different passages should be further explored in order to define the cause of variability. The use of undifferentiated equine primary myoblasts implies that metabolism and mitochondrial function may significantly differ compared with differentiated myotubes [30]. Therefore, it seems plausible to suspect that the oxidative phenotype, which depends on oxidative capacity and fiber type composition [30], would be impacted by myogenic differentiation and therefore is not completely reflected in situ metabolism. While the toxic effect of MCPA and MCPrG on ß-oxidation is well documented [9,10] it is worth noting that in this study as well as in the study performed by Lemieux et al. [13], the pathologic pathway is not restricted to an inhibition of ß-oxidation since SUIT1 and SUIT2 both result in a severe depression of the OXPHOS and ETS homeostasis. 

The concentrations used in the cytotoxicity/viability study ranged from 1 to 15 mM MCPA and from 0.25 to 1 mM HGA. Regarding the latter, no effect was observed at the aforementioned concentrations. This might indicate a lack of metabolization by the cell culture used within the time of the experiment. When analyzing both cytotoxicity and cell reduction capacity, it appears that a wider range of concentration must be tested in future experiments. Indeed, at 8 mM MCPA, cell reduction capacity, which is cumulative, increases. This may be imputed to the immediate effect of MCPA on the cells. It is, however, unclear if only some cells are less impacted by low concentrations of MCPA. In any event, at the lowest concentration after several hours the reductive capacity of the cells is restored and similar to control cells. However, it is clear that even at 1 mM, there is an immediate reaction to the toxin indicating cellular impairment. Also, best-fit value calculated for IC50 in our cytotoxicity experiment was 15.7 mM. This concentration would indicate that almost two times more MCPA is necessary to induce a cytotoxic effect compared to an effect on cell reductive capacity. When analyzed in detail, the calculated cytotoxicity IC50 value for passages 6 and 7 is 9.02 mM (±0.58 mM), similar to the IC50 obtained for the reducing potential. This raises the question of the fluctuation of cell types and maturity between passages as well as the sensitivity of these assays for these types of cells. Concentrations of MCPA (i.e., 9 mM) induced a severe decrease in mitochondrial respiration by HRR, compatible with a 50% decrease depending on the evaluated mitochondrial complex. However, the range of concentrations needs to be larger in future experiments in order to determine the minimal concentration at which a toxic effect of MCPA can be recorded. Also, despite a similar cell count, total protein content varied between runs. The differences between passages may also be related to a variable response on the cellular level to the toxin. 

In any case, our study manages to replicate mitochondrial alterations in response to MCPA intoxication. However, current mitochondrial activity assessment still relies on end-point assays, which yield limited kinetic and therefore prognostic information [31]. Indeed, our assays are based on oxygen flux recordings depending on substrates, pathways, and oxidative phosphorylation. Since in this case the clinical picture is a myopathy secondary to poisoning, it is essential to determine if the mitochondrial damage is consequential to the ß-oxidation defect and therefore toxic lipid accumulation. To determine if altered oxidative phosphorylation/mitochondrial respiration is causal or consequential to the clinical symptoms observed in AM affected horses, tissue-tissue interactions might need to be monitored to detect early if onsets of mitochondrial stress precede acute rhabdomyolysis. Even though cellular adaptations might be far-fetched and unrealistic in such an acute disease, defining the onset of stress in the first affected tissue will enhance chances of therapy. Since the mainly affected muscle fibers are oxidative, a mitochondrial dysfunction leading to a shift from oxidative phosphorylation to glycolysis, these cells may be less equipped to assume their role because of their limited ability to generate ATP by alternative means or because of the ultrastructural mitochondrial changes [19]. 

So far, many factors have been cited as potential contributors in the pathophysiology of mitochondrial dysfunction involved in a wide variety of disorders as decreased mitochondrial content, altered substrate delivery, muscle inflammation, morphological distortion of mitochondria due to glycogen cytoplasmic accumulation, oxidative damage and mitochondrial damage induced by gluco- and lipotoxicity secondary to intracellular substrate accumulation [32,33,34]. Since the direct effect of MCPA can be replicated on a cellular model, a down-regulation of nuclear and mitochondrial genes in AM does not seem plausible. However, the larger scale consequences on organs and organelles of HGA and MCPrG metabolization are to date unknown and may also constitute a therapeutic target strategy. Indeed, if the toxins have an impact on mitochondrial proteostasis, the damage may occur at different scales; the horse’s whole metabolism can be impacted, the mitochondrion’s interaction with the cell and the mitochondrion itself may be damaged, which will activate pathways to counteract the damage [35]. 

In the same line, MCPA-carnitine concentrations quantified in serum of AM-affected horses went up to several thousand nmol/L [7,15]. It is therefore imperative to compare our results to a direct dosage of MCPA-carnitine in muscle of AM affected horses as well as to more sensitive techniques, able to detect event slight augmentations of low concentrations. Additionally, purchased cells originated from one donor horse, and the reaction of these cells are therefore not to be extrapolated to all animals susceptible to HGA and MCPA intoxication. Through an immortal cell line, an easy-to-use and alternative can be found [36,37], providing a pure population of cells to reproduce results obtained in this preliminary study. A standardized cell culture with an immortal cell line will also minimize horse-associated reactions to the toxin as well as passage-dependent responses. 

In conclusion, our cellular in vitro model reproduced MCPA linked toxicity to a certain extend. For result reproduction, cytotoxicity assessment and in fine high throughput screening of therapeutic molecules, the use of an immortalized cell line is the next step. 

## Figures and Tables

**Table 1:** Substrate-uncoupler-inhibitor titration (SUIT) protocols performed.

	SUIT Protocols
SUIT1 ^1^	1GM; 2D; 3S; 4U; 5Rot; 6Ama
SUIT2 ^1^	1PM; 2D; 3G; 4S; 5U; 6Rot; 7Ama
SUIT3 ^2^	1ActM; 2D; 3S; 4U; 5Rot; 6Ama
SUIT4 ^2^	1OctM; 2D; 3S; 4U; 5Rot; 6Ama
SUIT5 ^2^	1PalM; 2D; 3S; 4U; 5Rot; 6Ama

^1^ NS-pathway. ^2^ F-pathway and S-pathway. Abbreviations: GM = Glutamate & malate; PM = Pyruvate & malate; ActM = Acetylcarnitine & malate; OctM = Octanoylcarnitine & malate; PalM = Palmitoylcarnitine & malate; D = ADP; G = Glutamate; S = Succinate; U = Uncoupler (FCCP); Rot = Rotenone; Ama = Antimycine A.

**Table 2:** Respirometric value percentage of MCPA treated cells compared to control cells.

Protocol	CI*_P_*	F*_P_*	CI + II*_P_*	CI + II*_E_*	CII*_E_*
SUIT 1	46%	-	43%	40%	32%
SUIT 2	53%	-	43%	40%	29%
SUIT 3	-	67%	61%	72%	51%
SUIT 4	-	74%	62%	56%	49%
SUIT 5	-	67%	60%	58%	37%

**Table 3:** Total protein content (±SEM) at each passage performed in duplicate (n = 2). Protein concentration is expressed in µg/µL).

P5	P6	P7	P8
4.17 ± 0.44	4.89 ± 0.55	3.79 ± 0.32	3.85 ± 0.12

## Data Availability

Equine primary myoblasts were obtained from RevaTis and are commercially available.
